# Predicting circRNA-drug sensitivity associations via graph attention auto-encoder

**DOI:** 10.1186/s12859-022-04694-y

**Published:** 2022-05-04

**Authors:** Lei Deng, Zixuan Liu, Yurong Qian, Jingpu Zhang

**Affiliations:** 1 School of Software, Xinjiang University, Urumqi, China; 2 School of Computer Science and Engineering, Central South University, Changsha, China; 3 School of Computer and Data Science, Henan University of Urban Construction, Pingdingshan, China

**Keywords:** circRNA-drug associations, Graph attention auto-encoder, Neural network, Similarity network

## Abstract

**Background:**

Circular RNAs (circRNAs) play essential roles in cancer development and therapy resistance. Many studies have shown that circRNA is closely related to human health. The expression of circRNAs also affects the sensitivity of cells to drugs, thereby significantly affecting the efficacy of drugs. However, traditional biological experiments are time-consuming and expensive to validate drug-related circRNAs. Therefore, it is an important and urgent task to develop an effective computational method for predicting unknown circRNA-drug associations.

**Results:**

In this work, we propose a computational framework (GATECDA) based on graph attention auto-encoder to predict circRNA-drug sensitivity associations. In GATECDA, we leverage multiple databases, containing the sequences of host genes of circRNAs, the structure of drugs, and circRNA-drug sensitivity associations. Based on the data, GATECDA employs Graph attention auto-encoder (GATE) to extract the low-dimensional representation of circRNA/drug, effectively retaining critical information in sparse high-dimensional features and realizing the effective fusion of nodes’ neighborhood information. Experimental results indicate that GATECDA achieves an average AUC of 89.18% under 10-fold cross-validation. Case studies further show the excellent performance of GATECDA.

**Conclusions:**

Many experimental results and case studies show that our proposed GATECDA method can effectively predict the circRNA-drug sensitivity associations.

## Background

Circular RNAs (circRNAs) are a new type of non-coding RNA produced by reverse splicing of introns, exons, or intergenic regions. CircRNA is more stable than linear RNA due to the absence of a covalent closed-loop structure and free terminal. Circular RNA is widely expressed in the human body, and its expression level is more than 10 times that of the corresponding linear mRNA. Recent studies have reported that circRNAs significantly affect the drug sensitivity of cells. For example, the expression of circAKT3 is high in cisplatin-resistant gastric cancer cells, and circ-PVT1 can promote the paclitaxel resistance of gastric cancer cells [1, 2]. In contrast, the high expression level of circCELSR1 can make ovarian cancer cells less sensitive to paclitaxel [3]. In addition, circSMARCA5 can increase the sensitivity of human breast cancer cells to cisplatin and bleomycin [4]. Therefore, identifying the circRNA-drug sensitivity associations is essential for circRNA-based therapy and drug discovery.

Traditional biological experiments take a long time and cost a lot. Efficient and accurate computational methods can significantly reduce the time and resources consumed by traditional biological research in predicting circRNA-drug sensitivity associations experiments. However, at present, researchers have done little work in this critical direction. Some studies in related fields have brought ideas for predicting circRNA-drug sensitivity associations. Chen et al. summarized some computational models which are used to identify miRNA-small molecule associations, and explained the development direction of computational methods for miRNA-small molecule association identification [5]. Moreover, Chen et al. proposed a new evaluation and validation for interaction prediction models [6]. In predicting gene regulatory networks and interactions, Liu et al. proposed the IMBDANET method to infer Gene Regulatory Networks based on the Improved Markov Blanket Discovery Algorithm [7]. Zhang et al. proposed the NDALMA model to predict lncRNA-miRNA Interactions by Network Distance Analysis [8]. In the prediction of small molecular-miRNA associations, Wang et al. proposed an EKRRSMMA model for predicting small molecule-miRNA associations based on ensemble of kernel ridge regression [9]. Chen et al. proposed a BNNRSMMA model for predicting potential small molecule-miRNA associations based on bounded nuclear norm regularization [10]. Recently, deep learning has been widely used in the field of association prediction and has achieved outstanding results. Peng et al. proposed a deep learning framework LPI-DLDN based on a dual-net neural architecture to find new associations of lncRNA-protein interactions [11]. LPI-DLDN integrates various biological features and can effectively reduce prediction errors. Zhou et al. proposed a gradient-boosting decision trees-based multi-layer framework LPI-deepGBDT to identify lncRNA-protein interactions [12]. Zhou et al. proposed a hybrid framework LPI-HyADBS to predict lncRNA-protein interactions [13]. LPI-HyADBS integrates multiple classification models, including deep neural networks, XGBoost, and SVM models with misclassification penalty coefficients. In the manuscript, we propose a new computational framework to predict the circRNA-drug sensitivity associations, hoping to improve the development efficiency of discovering circRNA-related drugs.

Fortunately, the circRic database systematically describes circRNA expression profiles in 935 cancer cell lines across 22 cancer lineages from Cancer Cell line Encyclopedia (CCLE) and furtherly analyzes the influence of circRNAs profile on drug sensitivity [14]. These data allow us to identify circRNA-drug sensitivity associations by computational methods.

In this study, we propose GATECDA, which is based on Graph Attention Auto-encoder(GATE) [15], to infer the circRNA-drug sensitivity associations. First, we curate the sequences of host genes of circRNAs, drug structure data and the circRNA-drug sensitivity associations, then calculate the circRNA similarities and drug similarities, respectively. Second, we generate the low-dimensional vector representations of the circRNA and drug nodes through GATE. Finally, we build a fully connected neural network, in which the vector representations are used as inputs, to make predictions of unknown associations. In the 5-fold and 10-fold cross-validation, GATECDA achieves the average area under the curve (AUC) of 89.18% and 88.45%, respectively. The results indicate that the GATECDA model we proposed can effectively predict circRNA-drug sensitivity associations.

At the same time, because drugs structure dramatically affects drugs function, we also use the structure information of drugs.

## Methods

### Dataset

In this work, we download the circRNA-drug sensitivity associations from the circRic [14] database, in which the drug sensitivity data comes from the GDSC database [16], containing 80076 associations that involve 404 circRNAs and 250 drugs. The circRic database systematically characterizes circRNAs expression profiles in 935 cancer cell lines across 22 cancer lineages from Cancer Cell line Encyclopedia, and analyzed the circRNAs biogenesis regulators, the effect of circRNAs on drug response and association between circRNAs with mRNA, protein, and mutation, and predicted RNA regulatory element in circRNAs. For each individual circRNA, the Wilcoxon test is applied to identify drug sensitivity which is significantly associated with the circRNAs expression. Meanwhile, the association with a false discovery rate (FDR) less than 0.05 is defined as a significant association. In our method, only these significant associations are extracted as a training set which includes 4134 associations involving 271 circRNAs and 218 drugs. We finally construct an association matrix $A \in R^{271\times 218}$ between circRNAs and drugs based on these significant associations. In *A*, element $A_{ij}=1$ indicates that circRNA and drug sensitivity are interrelated; otherwise, $A_{ij}=0$. Here, *i* and *j* denote the index of circRNA and drug in *A*, respectively. Besides the circRNA-drug sensitivity associations, we also curate the sequences of host genes of circRNAs and structure data of drugs, which come from the National Center for Biotechnology Information (NCBI) Gene database and PubChem database of NCBI, respectively [17, 18]. According to the sequences of host genes and structural information of drugs, their similarities are respectively calculated.

### Similarity networks

#### Sequence similarity of host genes of circRNAs

We calculate the sequence similarity between host genes as the similarity of circRNAs. The similarities are computed based on the Levenshtein distance of sequences through the ratio function of Python’s Levenshtein package. In the work, sequence similarities are represented by matrix $CSS\in R^{271\times 271}$.

#### GIP kernel similarity of circRNA

The GIP (Gaussian interaction profile) kernel similarity is widely used in the similarity calculation of biological entities in previous research [19]. Similarly, we calculate the GIP kernel similarity of circRNAs according to the circRNA-drug sensitivity associations matrix *A* based on the assumption that circRNAs associated with the same drug sensitivity are more likely to be similar. The GIP kernel similarity matrix of circRNAs is denoted by $CGS \in R^{271\times 271}$.

#### Structural similarity of drug

Since drugs structure dramatically affects drugs function, we can measure the similarity of drugs through their structures. Based on past studies, we chose the RDKit toolkit and the Tanimoto method to calculate the structural similarity of drugs [20, 21]. After obtaining these structure data from the PubChem database, we first used RDKit to calculate the topological fingerprint of each drug, then calculate the structure similarity between drugs through the Tanimoto method. Finally, the structure similarity matrix of drug is derived, denoted by $DSS\in R^{218\times 218}$.

#### GIP kernel similarity of drug

Similar to circRNA, we also calculate the GIP kernel similarity of drugs, which is represented by $DGS\in R^{218\times 218}$.

#### Similarity fusion method

As described above, we respectively calculate the similarities of circRNAs and drugs from different aspects. To obtain their comprehensive similarity matrix, the similarities from different aspects need to be fused. The circRNA’s comprehensive similarity matrix is constructed as follows.

1 $$\begin{aligned} \begin{aligned} CS_{ij}=\left\{ \begin{array}{lll} \frac{(CSS_{ij}+CGS_{ij})}{2} &{},&{} if\quad CSS_{ij} \ne 0\\ CGS_{ij} &{},&{} otherwise \end{array}\right. \end{aligned} \end{aligned}$$

Similarly, the drug’s comprehensive similarity matrix is computed as follows.

2 $$\begin{aligned} \begin{aligned} DS_{ij}=\left\{ \begin{array}{lll} \frac{(DSS_{ij}+DGS_{ij})}{2} &{},&{} if\quad DSS_{ij} \ne 0\\ DGS_{ij} &{},&{} otherwise \end{array}\right. \end{aligned} \end{aligned}$$

After obtaining the similarity networks, we binarize the similarity network for the downstream GATE model. In this step, we set the thresholds *cth* and *dth* for the binarization of circRNA similarity network and drug similarity network, respectively. We set the element in the similarity matrix to 1 if its value is greater than the threshold, otherwise 0.

### GATECDA framework

**Fig. 1:**
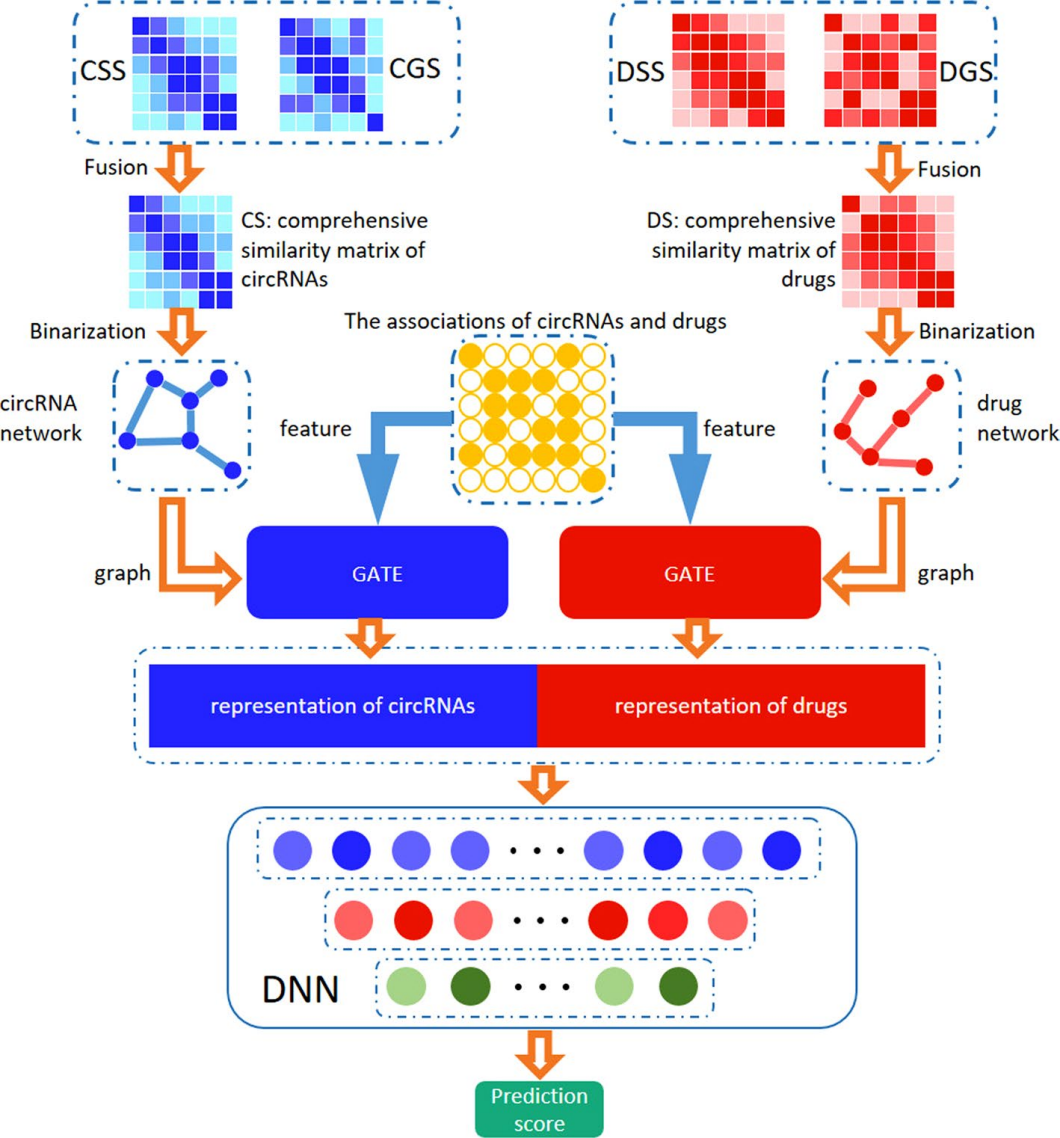
The flowchart of GATECDA. (1) We build a comprehensive similarity matrix CS for circRNAs and a comprehensive similarity matrix DS for drugs, respectively. (2) Two thresholds are utilized to binarize the corresponding comprehensive similarity matrices CS and DS. (3) GATE is employed to extract the representations of circRNAs and drugs respectively. (4). The representations of circRNAs and drugs are combined and fed into a fully connected neural network to predict the associations of each pair of circRNA and drug sensitivity

Our GATECDA model, the flowchart of which is depicted in Fig. 1, is based on Graph Attention Auto-encoder. The primary processing is composed of several steps: (1) Construct the circRNA and drug similarity network, respectively; (2) GATE is adopted to extract the vector representations of circRNAs and drugs; (3) The representations of circRNAs and drugs are combined and fed to a fully connected neural network for predicting the association score of each pair of circRNA and drug sensitivity.

### Graph attention auto-encoder

Graph Attention Auto-encoder(GATE) is an unsupervised learning model used for representation learning of structured Graph data. GATE can reconstruct node attributes and graphical structures of structured Graph data by stacking encoders and decoders. In the encoder, the attributes of nodes are fed into the encoders as the initial representation of nodes, and each encoder generates new representations of nodes by considering their relations based on a self-attention mechanism [22]. Furtherly, the encoder updates the representation of the current node with neighbors’ representations. In the decoder, the encoding process is reversed to reconstruct the initial attributes of nodes.

In this study, we used the GATE model to extract the representation of circRNAs and drugs. GATE assigns different weights to each neighbor of the current node through the attention mechanism, which can help the model to obtain better node representation.

**Fig. 2:**
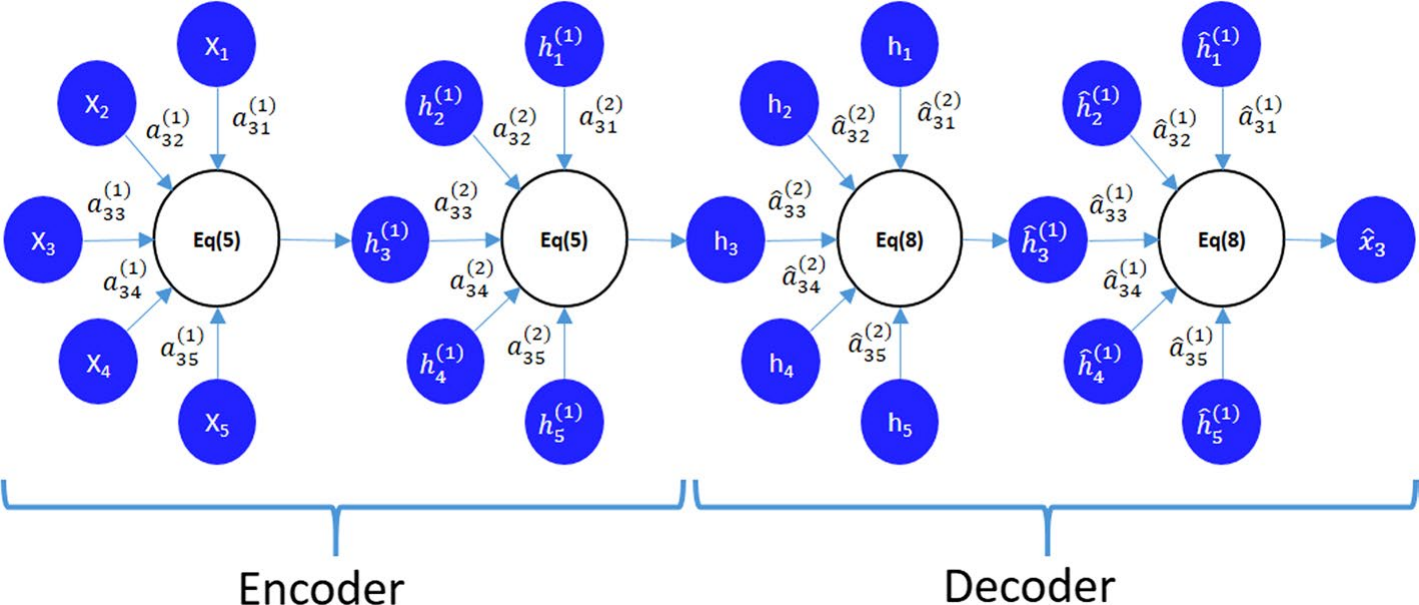
The process of using two-layer GATE to reconstruct the features of node 3. the neighbors of the node 3 are (1, 2, 3, 4, 5). we note that $h^{(0)}_i=x_i, h_i=h^{(2)}_i={\hat{h}}^{(2)}_i$ , and ${\hat{x}}_i={\hat{h}}^{(0)}_i$, $\forall i \in {1,2,\ldots ,N}$

The GATE model consists of multiple encoder layers and decoder layers. In GATE, encoders and decoders have the same number of layers. The multiple encoder layers can improve the learning ability of the model and produce a better node representation. Figure 2 shows the process of GATE encoding and decoding.

The Encoder layer generates new representations for nodes by taking into account their neighbors’ representations based on their relevance. Inspired by the work of Velickovic et al. [22], the GATE model employs a self-attention mechanism with shared parameters among nodes to determine the relations between one node and its neighbors. In the *k*th layer encoder, the correlation between node *i* and its neighbor node *j* is calculated as follows:

3 $$\begin{aligned} \begin{aligned} c^{(k)}_{ij}=Sigmoid( V_s^{(k)^{T}} \sigma (W^{(k)}h_i^{(k-1)}) + V_r^{(k)^{T}} \sigma (W^{(k)}h_j^{(k-1)}) ) \end{aligned} \end{aligned}$$

Here, ${W^{(k)}} \in {R^{{d^{(k)}} \times {d^{(k - 1)}}}}$, $V_s^{(k)}\in R^{d^{(k)}}$, and $V_r^{(k)}\in R^{d^{(k)}}$ are the trainable parameter of the *k*th layer encoder, $\sigma$ and Sigmoid represent the activation function and the Sigmoid function, respectively.

To solve the problem of comparability among coefficients of node *i*’s neighbors, we employ the Softmax function to normalize the coefficients as shown in the following Equ. (4):

4 $$\begin{aligned} \begin{aligned} \alpha _{ij}^{(k)}= \frac{exp(c_{ij}^{(k)})}{\sum _{l\in N_i}exp(c_{il}^{(k)})} \end{aligned} \end{aligned}$$

where $N_i$ denotes the neighbors of node *i*, including node *i* itself.

The node features are taken as initial node representations, namely $h_i^{(0)}=x_i$, and then the representation of node *i* in the *k*th layer is generated by the Eq. (5):

5 $$\begin{aligned} \begin{aligned} h_i^{k}=\sum _{j\in N_i} \alpha _{ij}^{(k)} \sigma (W^{(k)}h_j^{(k-1)}) \end{aligned} \end{aligned}$$

The last encoder layer’s output will be considered as the node representations used in our model.

GATE unsupervised learn node representations through utilizing the same number of decoder layers as the encoder. Each decoder layer reconstructs the representations of nodes according to the representations of their neighbors based on their relevance. The normalized relevance between node *i* and a neighbor *j* in the *k*th layer decoder is calculated by the Eq. (6) and (7).

6 $${\hat{\alpha }}_{ij}^{(k)}=\frac{exp({\hat{c}}_{ij}^{(k)})}{\sum _{l\in N_i}exp({\hat{c}}_{il}^{(k)})}$$

7 $${\hat{c}}^{k}_{ij}=Sigmoid( {\hat{v}}_s^{(k)^{T}} \sigma ({\hat{W}}^{(k)} {\hat{h}}_i^{(k)}) + {\hat{v}}_r^{(k)^{T}} \sigma ({\hat{W}}^{(k)}{\hat{h}}_j^{(k)}) )$$

Similar to the encoder layers, ${\hat{W}}^{k}\in R^{d^{(k)}\times d^{(k-1)}}$, ${\hat{v}}_s^{(k)}\in R^{d^{(k-1)}}$, and ${\hat{v}}_r^{(k)}\in R^{d^{(k-1)}}$ are also the trainable parameters of the *k*th layer decoder. The input of the decoder comes from the output of the last layer encoder, and the *k*th decoder will reconstruct the node representation of layer k-1 according to the Eq. (8).

8 $$\begin{aligned} \begin{aligned} {\hat{h}}_i^{k-1}=\sum _{j\in N_i} {\hat{\alpha }}_{ij}^{(k)} \sigma ({\hat{W}}^{(k)}{\hat{h}}_j^{(k)}) \end{aligned} \end{aligned}$$

After decoding via L decoder layers, the last decoder layer’s output is considered the reconstructed node features.

The loss function consists of two parts, namely the reconstruction loss of node features and the reconstruction loss of graph structure. We combine them through the equation as follows:

9 $$\begin{aligned} \begin{aligned} Loss=\sum ^{N}_{i=1}||x_i-\hat{x_i}||_2-\lambda \sum _{j\in N_i}log\left( \frac{1}{1+exp(-h^T_ih_j)}\right) \end{aligned} \end{aligned}$$

Here, $\lambda$ is a hyperparameter, which balances the contribution of reconstruction loss of graph structure. $x_i$ and $\hat{x_i}$ represent the node features and the reconstructed features of nodes respectively. $h_j$ is the representation of a neighboring node *j* to node *i*. We can obtain high-quality node representations by minimizing the Loss function.

## Results and discussion

### Evaluation metrics

In this work, we evaluate the predictive performance of our method by employing 5-fold and 10-fold cross-validation (CV). During the evaluation, we randomly divide all circRNA-drug sensitivity associations into 5 folds or 10 folds, one of which is used as a test set and the other as a training set. Then, we draw the Receiver Operating Characteristics (ROC) curve and calculate the area under the ROC curve (AUC) to quantify the performance of the approach. In order to comprehensively assess the method, we also utilize the $F_1$ score, accuracy, recall, specificity, precision, and area under the accuracy-recall curve (AUPR) to evaluate the performance.

### Parameters tuning

Different parameter values will affect the prediction performance of GATECDA. There exist numerous hyperparameters to be tuned, and they can be divided into three parts: the parameters in GATE, the parameters in the classifier (a fully connected neural network), and the cutoffs in binarization.

Optimizable hyperparameters in GATE:

i The number of layers. According to the research in GATE, the number of layers of the encoder and decoder are both set to 2.
ii the number of neurons in each layer. The decoder layer has the same number of neurons as the corresponding encoder layer. There are 128 and 64 neurons in the two encoder layers, respectively.
iii Learning rate. We select the learning rate of GATE in {$10^{-2},10^{-3},10^{-4}$}. When we set the learning rate to $10^{-2}$ or $10^{-4}$, it will be difficult or slow for the loss of GATE to converge. When the learning rate is $10^{-3}$, GATE can quickly reach the state of convergence. Based on the above results, we set the learning rate of GATE to $10^{-3}$.
iv *lambda* and *dropout*. *lambda* controls the contribution of graph structure reconstruction in the loss function. *dropout* refers to temporarily dropping out network units from the network during training in a certain probability. These two parameters have no significant impact on the performance of the model. We set *lambda* and *dropout* to their default values of 1 and 0.

Optimizable hyperparameters in classifier:

i The number of layers and the number of hidden neurons in each layer. The output of GATE is fed into the classifier, which is implemented by a neural network. We utilize a classical three-layer neural network architecture, which contains 128, 64, and 32 neurons, respectively.
ii Optimizer and learning rate. Adam optimizer is employed in the classifier and the initial learning rate is set to $10^{-4}$.
iii Initial values of weights and biases. The Glorot uniform distribution initializer is employed to initialize the weights, and the biases are initialized to 0.

**Fig. 3:**
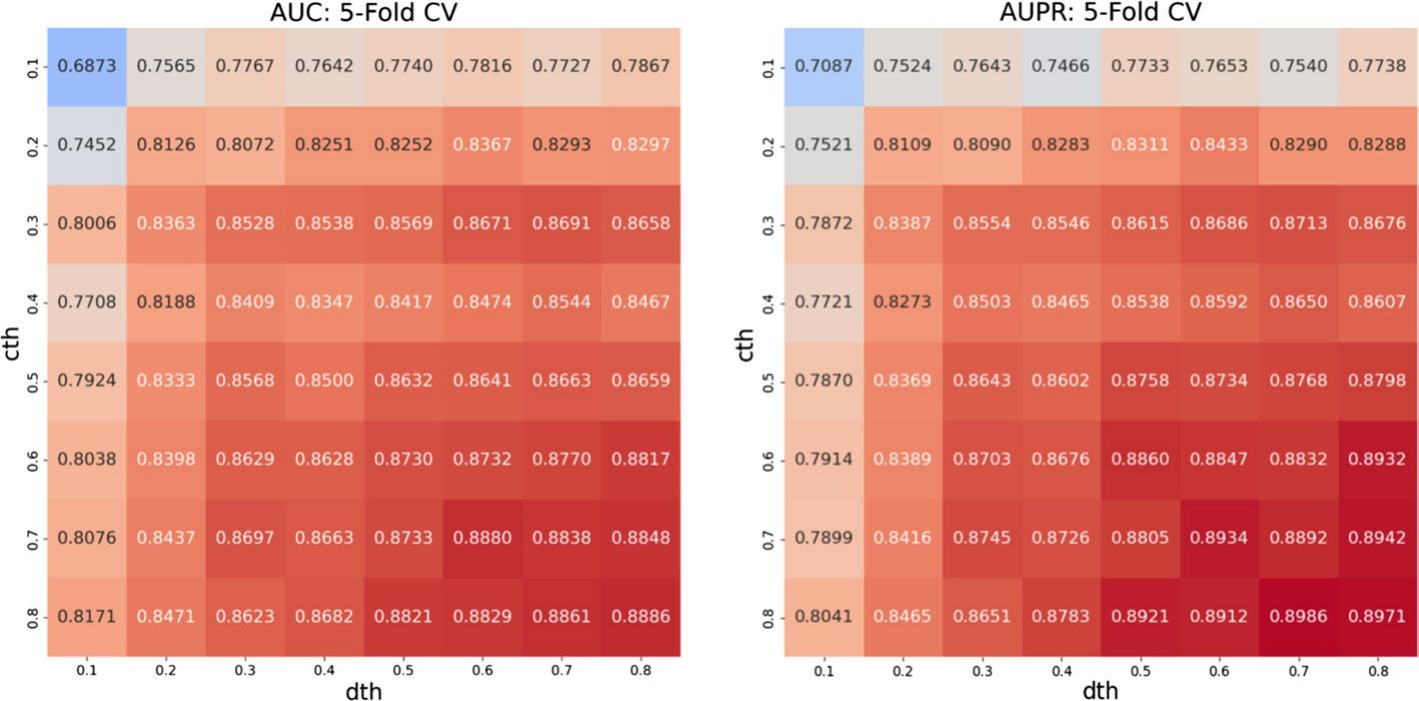
Performance with different combinations of the two hyperparameters

Optimizable hyperparameters in binarization:

i threshold (*cth*) and (*dth*). In our method, *cth* and *dth* are the cutoffs for the binarization of circRNA similarity network and drug similarity network, respectively. The two parameters are tuned using 5-fold cross-validation through grid search. As shown in Fig. 3, the model’s performance is gradually improved with the increase of *cth* and *dth*. Moreover, when *cth* and *dth* reach 0.7 and 0.6, AUC and AUPR will converge. A higher threshold can effectively reduce the noise in the similarity network, but it will eliminate the practical information in the similarity network. In order to ensure that there is more helpful information in the similarity network, we consider it is more appropriate to set *cth* and *dth* to 0.7 and 0.6, respectively.

### The benefits of merging multiple similarity networks

In order to compare the effect of single similarity and fusion similarity on model As mentioned above, not only the circRNA-drug sensitivity associations but also the sequences of host genes of circRNAs and structural information of drugs are integrated into our GATECDA method. To examine the effect of considering the multiple similarity networks, we test GATECDA on four different network configurations:

- **GATECDA-S**: The global network consisting of Sequence similarity network of host gene of circRNAs and Structure similarity network of drugs.
- **GATECDA-G**:The global network consisting of GIP kernel similarity network of circRNAs and GIP kernel similarity network of drugs.
- **GATECDA-SNF**: This global network consisting of comprehensive similarity network of circRNAs and drugs, which are built through the SNF methods [23], respectively.
- **GATECDA**: This global network consisting of comprehensive similarity network of circRNAs and drugs, which are built through the arithmetic average strategy according to the formulas (1) and (2), respectively.

**Fig. 4:**
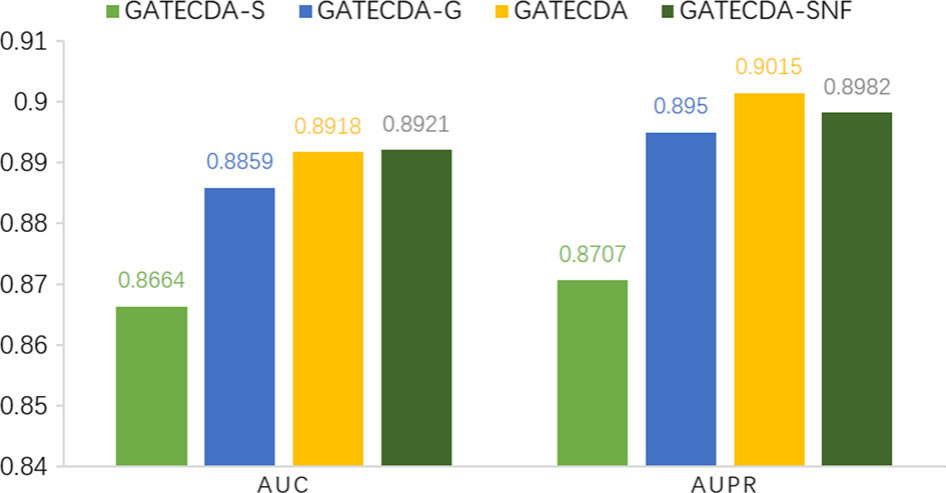
Comparison on different network configurations in terms of AUC and AUPR

The comparison based on 10-fold CV is carried out and the results are shown in Fig. 4 and Table 1. The AUC and AUPR scores are 0.8918 and 0.9025 for GATECDA in Fig. 4 respectively. The $F_1$ score is 0.8234 for GATECDA in Table 1. GATECDA-SNF achieves the similar results of 0.8921, 0.8982 and 0.8236 on the three evaluation metrics respectively. Clearly, the results show that GATECDA and GATECDA-SNF both outperform GATECDA-S and GATECDA-G. The two models can benefit from merging multiple similarity networks.

**Table 1:** Comparison on different network configurations in terms of $F_1$ score, accuracy, recall, specificity and precision using 10-fold cross-validation

Methods	Precision	Recall	F1 score	Accuracy	Specificity
GATECDA-S	0.7576	0.8468	0.7997	0.7879	0.7290
GATECDA-G	0.7896	**0**.**8500**	0.8187	0.8118	0.7735
GATECDA-SNF	**0**.**8138**	0.8335	**0**.**8236**	**0**.**8214**	**0**.**8093**
GATECDA	0.8128	0.8343	0.8234	0.8211	0.8079

The values with bold indicate the best results in terms of different metrics

GATECDA-SNF gains the performance comparable to that of GATECDA. However, the SNF method requires more computations compared to that of the arithmetic average strategy. Therefore, in GATECDA, we still choose the arithmetic mean strategy which is more convenient to calculate the similarity fusion.

### Comparison with other methods

To our knowledge, there are few models for predicting the circRNA-drug sensitivity associations. Therefore, we compare the GATECDA model with four models that address other association prediction tasks in the bioinformatics field, including one classic method and three state-of-the-art models. Among the four methods, the KATZ measure [24] is a classic network-based method to calculate the similarity between nodes in a heterogeneous network. The other three methods are all developed based on GNN. VGAE [25] and VGAMF [26] are used to predict the associations between miRNA and disease. The GCNMDA [27] model is used to predict the associations between microbes and drugs.

**Fig. 5:**
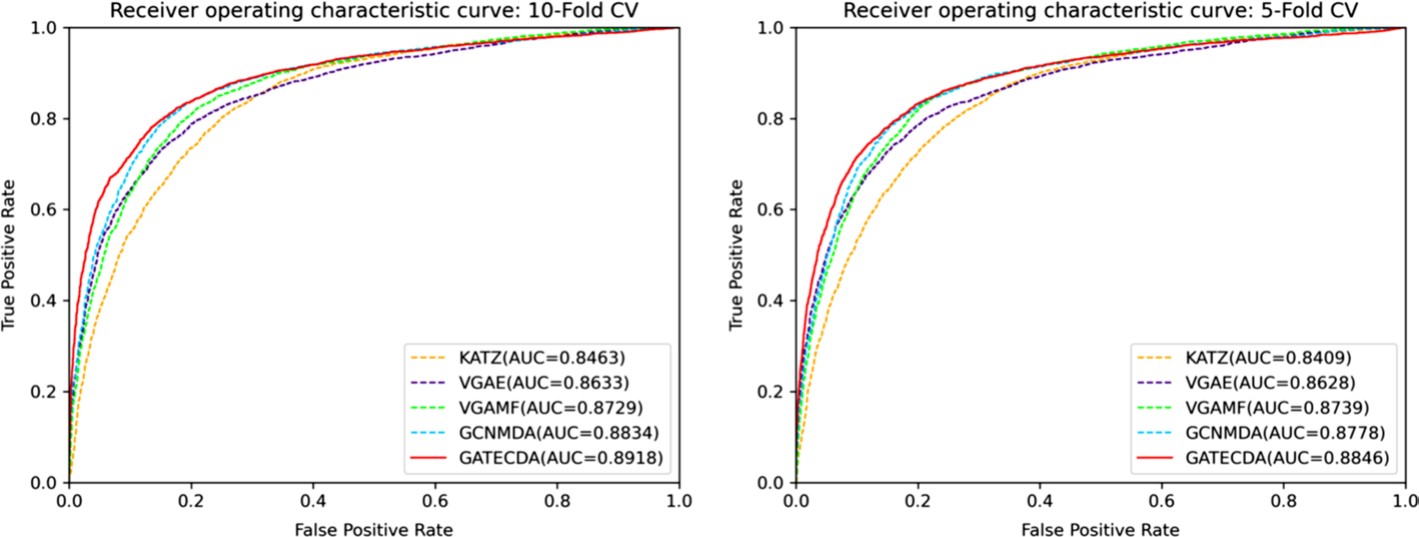
Comparison results of GATECDA with the four state-of-the-art methods using 10-fold CV and 5-fold CV

The best parameter values in each comparison method are set according to the authors’ recommendation in their papers. To perform a fair comparison, these methods are carried out by employing the same data. Among all the methods, our GATECDA model gains the best prediction performance. Figure 5, Tables 2 and 3 depict the experimental results of GATECDA and the four comparison methods in terms of 10-fold CV and 5-fold CV. In 10-fold cross-validation, the average AUC and AUPR of GATECDA reach 0.8918 and 0.9015, respectively. Following GATECDA, the GCNMDA method obtains 0.8834 and 0.8864 in terms of AUC and AUPR, respectively. In addition, we also compare these methods in terms of other metrics including precision, recall, $F_1$, accuracy and specificity. GATECDA almost outperforms the other four methods except that the recall is relatively lower. Considering that the $F_1$ score can more comprehensively reflect the model’s performance, the results in Table 2 show that the overall performance of GATECDA is still the best.

**Table 2:** Comparison with the four state-of-the-art methods in terms of aupr, $F_1$ score, accuracy, recall, specificity and precision using 10-fold cross-validation

Methods	AUPR	Precision	Recall	F1 score	Accuracy	Specificity
KATZ	0.8269	0.7176	**0**.**8800**	0.7906	0.7669	0.6538
VGAE	0.8725	0.7683	0.8313	0.7986	0.7864	0.7398
VGAMF	0.8681	0.7783	0.8471	0.8113	0.8029	0.7588
GCNMDA	0.8864	0.8039	0.8420	0.8225	0.8183	0.7946
GATECDA	**0**.**9015**	**0**.**8128**	0.8343	**0**.**8234**	**0**.**8211**	**0**.**8079**

The values with bold indicate the best results in terms of different metrics

**Table 3:** Comparison with the four state-of-the-art methods in terms of aupr, $F_1$ score, accuracy, recall, specificity and precision using 5-fold cross-validation

Methods	AUPR	Precision	Recall	F1 score	Accuracy	Specificity
KATZ	0.8223	0.7141	**0**.**8756**	0.7866	0.7625	0.6494
VGAE	0.8730	0.7763	0.8226	0.7988	0.7892	0.7546
VGAMF	0.8661	0.7911	0.8437	0.8165	0.8104	0.7772
GCNMDA	0.8761	0.7938	0.8427	0.8175	0.8119	0.7810
GATECDA	**0**.**8928**	**0**.**8076**	0.8316	**0**.**8194**	**0**.**8167**	**0**.**8018**

The values with bold indicate the best results in terms of different metrics

The result in Table 3 is similar to that in Table 2. From the two tables, we can find that the results in terms of 10-fold CV are slightly better than those in terms of 5-fold CV. The improvement in results should be due to more data available in 10-fold CV than that in 5-fold CV during training. Hence, these results indicate that GATECDA is an effective method to predict the circRNA-drug sensitivity associations.

### Case studies

To further evaluate the predictive performance of the GATECDA method, we conduct case studies on two drugs: PAC-1 and Foretinib. The circRNA-drug sensitivity associations corresponding to drug sensitivity in GDSC database is used as the training set, and the circRNA-drug associations corresponding to drug sensitivity in CTRP is as the test set [28]. Among the predicted scores of associations between each drug and these circRNAs, we select the top 20 circRNAs with the highest scores.

The drug PAC-1 is a potent activator of Procaspase-3. PAC-1 acts on primary cancer cells and induces apoptosis. In cell culture, PAC-1 has produced cytotoxicity against various cancer cells, including lymphoma, multiple myeloma, and many others [29]. PAC-1 has been used in the trials studying for the treatment of Lymphoma, Melanoma, Solid Tumors, Breast Cancer, and Thoracic Cancers [30].

As shown in Table 4, among the top 10 predicted circRNAs related to PAC-1, there are 9 circRNAs which have been confirmed in circRic, and 17 of the top 20 have been confirmed.

**Table 4:** The top 20 circRNAs associated with drug PAC-1. circRic(CTRP) indicates that the drug sensitivity in one circRNA-drug association is derived from the CTRP database

Rank	circRNA	Evidence	Rank	circRNA	Evidence
1	VIM*	circRic(CTRP)	11	MEF2D*	circRic(CTRP)
2	CTTN*	circRic(CTRP)	12	PEA15*	circRic(CTRP)
3	POLR2A*	circRic(CTRP)	13	FBLN1*	circRic(CTRP)
4	CRIM1*	circRic(CTRP)	14	NCL	Nonsignificant
5	THBS1*	circRic(CTRP)	15	COL1A2*	circRic(CTRP)
6	ANP32B*	circRic(CTRP)	16	DCBLD2*	circRic(CTRP)
7	COL1A1*	circRic(CTRP)	17	COL6A2*	circRic(CTRP)
8	PTMS*	circRic(CTRP)	18	EHBP1L1	Nonsignificant
9	SPINT2	Nonsignificant	19	PSAP*	circRic(CTRP)
10	ASPH*	circRic(CTRP)	20	ANKRD36C*	circRic(CTRP)

Nonsignificant means non-significant association. circRNAs marked with ‘*’ are verified

Foretinib is an orally bioavailable small molecule with potential antineoplastic activity [31–33]. Foretinib inhibits tumor angiogenesis, proliferation, and metastasis by blocking the C-Met and VEGFR2 pathways [34]. Table 5 shows that 7 of the top 10 and 16 of the top 20 have been confirmed in circRic.

**Table 5:** The top 20 circRNAs associated with drug Foretinib

Rank	circRNA	Evidence	Rank	circRNA	Evidence
1	MUC16*	circRic(CTRP)	11	THBS1*	circRic(CTRP)
2	EVPL	Nonsignificant	12	PSAP*	circRic(CTRP)
3	ANP32B*	circRic(CTRP)	13	ARID1B*	circRic(CTRP)
4	ASPH*	circRic(CTRP)	14	WASF1*	circRic(CTRP)
5	GJB3*	circRic(CTRP)	15	LTBP3*	circRic(CTRP)
6	PTMS*	circRic(CTRP)	16	CRIM1*	circRic(CTRP)
7	CNKSR1*	circRic(CTRP)	17	MYC	Nonsignificant
8	LCN2*	circRic(CTRP)	18	ANKRD36C*	circRic(CTRP)
9	FBLN1	Nonsignificant	19	PLEKHG2*	circRic(CTRP)
10	PHF21A	Nonsignificant	20	ANXA2*	circRic(CTRP)

circRNAs marked with ‘*’ are verified

To evaluate the predictive performance of GATECDA for potential circRNAs relevant to new drugs, we select two drugs with only one known circRNA-drug association in the dataset for de novo testing. We remove the only association of these two drugs with circRNAs and consider them as new drugs. They are erlotinib and MG-132 respectively. Erlotinib is a tyrosine kinase receptor inhibitor commonly used in pancreatic or non-small cell lung cancer [35]. MG-132 is a tripeptide that acts as a proteasome inhibitor to alleviate DNA damage and apoptosis [36]. For new drugs without any known circRNA-drug associations, GATECDA can calculate its features through neighbor nodes in the network. Considering that the new drug has no circRNA-drug association, which will affect the calculation of the model, we choose to generate the initial features of new drug in the graph by random assignment. Finally, we evaluate the prediction results using circRNA-drug associations in the circRic database, in which drug sensitivity data are obtained from CTRP.

As shown in Table 6, 5 of the top 10 predicted circRNAs associated with erlotinib have been confirmed in circRic, and 4 of the top 10 circRNAs related to MG-132 have been confirmed in circRic.

**Table 6:** The top 10 predicted circRNAs related to two new drugs

	Erlotinib			MG-132	
Rank	circRNA	Evidence	Rank	circRNA	Evidence
1	SPINT2*	circRic(CTRP)	1	CRIM1	Nonsignificant
2	KRT19*	circRic(CTRP)	2	THBS1*	circRic(CTRP)
3	POLR2A	Nonsignificant	3	SPINT2	Nonsignificant
4	LTBP3	Nonsignificant	4	AHNAK	Nonsignificant
5	KRT7*	circRic(CTRP)	5	KRT19*	circRic(CTRP)
6	FN1	Nonsignificant	6	EFEMP1*	circRic(CTRP)
7	THBS1	Nonsignificant	7	COL1A2	Nonsignificant
8	MAL2*	circRic(CTRP)	8	ANXA2*	circRic(CTRP)
9	CRIM1	Nonsignificant	9	COL8A1	unconfirmed
10	LCN2*	circRic(CTRP)	10	COL6A2	Nonsignificant

circRNAs marked with ‘*’ are verified

## Conclusions

Recent studies have shown that circRNA plays an essential role in human health. Predicting the circRNA-drug sensitivity associations can advance the development and utilization of drugs, so as to help in the treatment of diseases. The computation-based approaches could accelerate the discovery of circRNA-drug sensitivity associations. In this manuscript, we propose GATECDA, an efficient computational method based on graph attention autoencoder, to predict circRNA-drug sensitivity associations. Many experimental results and case studies show that our proposed GATECDA method can effectively predict the relationship between circRNA and drug sensitivity. In the experiments of 5-fold CV and 10-fold CV, the AUC of GATECDA reaches 0.8846 and 0.8918, respectively. This result is superior to other comparable methods. Of course, the GATECDA model also has certain shortcomings. For example, when predicting circRNAs related to new drugs, because the new drugs do not have known associations with circRNAs in the dataset, this will lead to the cold start of the model. In predicting circRNAs related to new drugs, we choose to solve this problem by random assignment, but the effect is not particularly good. To address these issues and further improve the model performance. In subsequent studies, we will collect more circRNA-drug sensitivity associations and integrate more biological information to reduce the model’s reliance on known circRNA-drug associations, such as multiple circRNA-drug similarities and associations between circRNAs, drugs, and diseases. We will eliminate the model’s deficiencies in new drug prediction by enriching the data from various sources.

## Data Availability

The datasets were derived from sources in the public domain: the circRNA-drug sensitivity associations from https://hanlab.tamhsc.edu/cRic/, the sequences of host genes of circRNAs from https://www.ncbi.nlm.nih.gov/gene, the structure data of drugs from https://pubchem.ncbi.nlm.nih.gov/. The code and datasets of GATECDA is available at https://github.com/yjslzx/GATECDA.

## Abbreviations

GATE: Graph attention auto-encoder
PAC-1: Procaspase activating compound 1
CCLE: Cancer cell line encyclopedia
NCI: National Cancer Institute
NCBI: National Center for Biotechnology Information
GDSC: Genomics of Drug Sensitivity in Cancer
CTRP: Cancer Therapeutics Response Portal
FDR: False discovery rate
GNN: Graph neural network
GIP: Gaussian interaction profile
CSS: Sequence similarity of host gene of circRNAs
CGS: GIP kernel similarity of circRNA
CS: Comprehensive similarity matrix of circRNA
DSS: Structure similarity of drug
DGS: GIP kernel similarity of drug
DS: Comprehensive similarity matrix of drugs
BERT: Bidirectional Encoder Representation from Transformers
GAT: Graph attention networks
ROC: Receiver operating characteristics curve
AUC: The area under the ROC curve
TPR: True positive rate
FPR: False positive rate
AUPR: Area under the accuracy-recall curve
CV: Cross-validation
